# Breast Cancer Milieu Maneuvers Cancer-Associated Macrophages to Synergize Neoplastic Repertoires

**DOI:** 10.3390/cancers18101596

**Published:** 2026-05-14

**Authors:** Huey-Jen Lin, Yingguang Liu, Brooke Langevin, Jiayuh Lin

**Affiliations:** 1 Department of Medical & Molecular Sciences, University of Delaware, Willard Hall Education Building, 16 West Main Street, Newark, DE 19716, USA; 2 Department of Molecular and Cellular Sciences, College of Osteopathic Medicine, Liberty University, 306 Liberty View Lane, Lynchburg, VA 24502, USA; yliu@liberty.edu; 3 Department of Practice, Sciences and Health Outcomes Research, Center for Translational Medicine, School of Pharmacy, University of Maryland, 20 North Pine Street, Baltimore, MD 21201, USA; blangevin@umaryland.edu; 4 Department of Biochemistry and Molecular Biology, Molecular Medicine Graduate Program, University of Maryland School of Medicine and Greenebaum Comprehensive Cancer Center, 108 N. Greene Street, Baltimore, MD 21201, USA; jlin@som.umaryland.edu

**Keywords:** breast cancer, chemotherapy, cytokines, hypoxia, immune checkpoint blockades, immune suppression, M1, M2, oncometabolites, signal transduction cascades, tumor-associated macrophages, tumor microenvironment

## Abstract

Breast cancer is one of the most devastating malignancies in women worldwide. A growing body of evidence has linked neoplastic growth, invasion, metastasis, immune escape, and therapeutic resistance to infiltrating tumor-associated macrophages. In a breast cancer mass, macrophages are largely polarized to two main subtypes, M1 and M2, albeit with continuum intermediates, based on their immunological behaviors, gene signatures, and functional roles. While the former portrays proinflammatory and anti-cancer effects, the latter elicits the opposite impacts. M2 macrophages have gained rising attention as they are largely involved in fostering an immune-suppressive, cancer-promoting landscape and are imperative for malignant features across breast cancer subtypes. Through a positive feedback paracrine loop, M2 macrophages can be enriched by a plethora of dysregulated oncogenic signaling mediators, exemplified by CSF1/CSF1R, STAT3, IL-6, YAP, PI3K, PDK1, and AKT. These modulators could be released from or activated by surrounding malignant cells, fibroblasts, secreted extracellular vesicles, cell fragments generated after chemotherapies, hypoxia, dysregulated immune checkpoint pathways or oncometabolites. This review aims to discern the molecular cues fortifying M2 subpopulations. Moreover, recent advances in single-cell sequencing, spatial, and computational approaches have refined the understanding of TAM heterogeneity, while clinical translation remains limited by low therapeutic specificity, compensatory signaling, and differences between murine and human macrophage biology. Future therapeutic regimens should include strategies aimed at correcting aberrations that favor M2 polarization and are justified with divergences between humans and mice.

## Simple Summary

Breast cancer is the most commonly diagnosed malignancy in women. Research increasingly shows that cancer growth, spread to other parts of body, and resistance to therapeutic regimens are strongly influenced by the body’s immune system. One important group of immune cells involved is macrophages. Although macrophages normally help defend the body, the breast tumor microenvironment can change them into tumor-associated macrophages during cancer progression. Consequently, these macrophages can enhance cancer growth, increase metastasis, support new blood vessel formation, and hijack the host's naïve anti-tumor immunity. This article outlines various mechanisms employed by breast cancer cells and the tumor microenvironment to shift macrophages from their initial tumor-suppressing toward tumor-helping state. Variables involved in promoting cancer growth include low oxygen levels, abnormal metabolisms in sugar and fat, as well as broken cell debris following chemotherapeutics. This paper depicts preclinical laboratorial and animal studies as well as exemplified clinical trials. It presents the opportunities, challenges, and obstacles that entail macrophage-targeting therapies.

## 1. Introduction

### 1.1. Introduction of Breast Cancer

Breast cancer (BC) is the most frequently diagnosed malignancy in women globally. The global cancer observatory (GLOBOCAN) captures data from 185 territorial regions worldwide for 36 cancers subclassified by gender and by age. In 2022, BC not only accounted for approximately 2.30 million new cases, representing 11.5% of all cancer incidences, but also conveyed the highest cancer-related mortality in females with 0.666 million deaths [1]. Due to the molecular heterogeneity, BC is sub-categorized according to the presence or absence of estrogen receptors (ERs), the progesterone receptor (PR), and human epidermal growth factor receptor 2 (HER2), as well as by the percentage of the proliferating index Ki67. Consequently, BC is classified into five main subtypes: luminal A, luminal B HER2^−^, luminal B HER2^+^, HER2^+^, and triple-negative BC (TNBC) [2,3].

The luminal A subtype is characterized by the expression of ERs and/or PR with negligible HER2 and Ki67 below 14%, while luminal B presents a more aggressive profile than luminal A and is further divided into luminal B HER2^−^ and luminal B HER2^+^. The former is distinguished by the expression of ERs and/or PR with undetectable HER2 and with Ki67 greater than or equal to 14%, while the latter expresses ERs and/or PR, along with detectable HER2 regardless of Ki67 percentage. The HER2-enriched subtype lacks ERs and PR yet expresses abundant HER2 receptors with any Ki67 percentage. This subtype manifests a poorer prognosis than luminal subtypes, although targeted therapy implementing anti-HER2 antibodies have been developed [2]. Moreover, TNBC lacks receptors for ERs, PR, and HER2 and is associated with the worst prognosis with highest mortality among all subgroups, due to the high invasiveness, distant metastasis, and treatment resistance [2,3,4]. These molecular profiles have enabled the formulation of more precise and personalized treatment regimens [3,5]. Yet, TNBC and hormone therapy-resistance BC continue to arise, limit therapeutic efficacy, and yield high mortality [6,7]. Further studies have revealed that the pleiotropic tumor microenvironment promotes neoplastic features associated with poor prognosis [8].

### 1.2. Introduction of Tumor Microenvironment (TME) and Cancer-Associated Macrophages (TAMs)

The TME in BC consists of cancer cells, infiltrating immune cells such as macrophages and lymphocytes, as well as aberrant oncometabolites, immune checkpoint regulators, extracellular vesicles (EVs), immunosuppressive cytokines, epigenetic controllers, and signaling modulators. Together, they not only foster immune tolerance but also hinder anti-cancer cytotoxicity [9]. A growing body of research has been drawn to unveil the imperative role played by an immunosuppressive TME that augments distant metastasis and therapeutic resistance [10]. Tumor growth can fortify an influx of bone marrow-derived monocytes that differentiate into monocytic myeloid-derived suppressor cells (MDSCs) and TAMs [11,12,13]. Together, they foster an immunosuppressive and tumor-promoting TME. In BC, TAMs comprise the most abundant immune cell type in the TME [14,15], accounting for approximately 50% of the cells in the cancer mass [16,17,18]. They present a high degree of plasticity and heterogeneity to adapt to the ever-changing environmental cues [19,20,21,22,23].

In the early phase of BC, TAMs exert a proinflammatory anti-cancer phenotype (M1-like) and inhibit neoplastic progression by secreting reactive oxygen species (ROS) and tumor necrosis factor-α (TNF-α) or even by augmenting phagocytosis [24]. As BC exacerbates, cancer cells can secrete factors or release immunomodulatory cytokines encapsulated in EVs to hijack TAMs and favor macrophage polarization toward the M2-like phenotype, which manifests immunosuppressive and cancer-promoting features [25,26]. Via the secretion of interleukin (IL)-10 and transforming growth factor β (TGFβ), M2 macrophages attenuate tumoricidal effects exerted by CD8^+^ cytotoxic T lymphocyte (CTL) and natural killer cells (NKs) [24,27]. It is worth mentioning that progressive BC often expresses a continuum of M1 and M2 with varying ratios. The proximity of TAMs to the cancer mass influences their subtype distribution and function, with those closer to the tumor core typically exhibiting more M2 phenotypes, associated with worse prognosis and facilitating neoplastic growth, while those further away are predominantly M1 phenotypes and linked to a better prognosis [28,29,30]. M2-like macrophages play a central role in facilitating neoplastic growth, angiogenesis, metastasis, and immune escape through a plethora of mechanisms across BC subtypes [31,32,33].

Moreover, M2-like TAMs are further categorized into M2a, M2b, M2c, and M2d subtypes, based on the environmental stimuli they received, secreted enzymes or effectors, and their functional roles [31,34]. The M2a subgroup enhances the migratory and invasive capabilities of BC cells [35]. M2b contributes to bevacizumab resistance in TNBC [36]. The M2c subtype enhances the metastatic potential of BC cells and is significantly elevated in advanced stage II and III BC [37], while M2d macrophages promote angiogenesis and immune tolerance and support tumor growth in TNBC and HER2-overexpressing BC [34]. Nevertheless, this review aims to outline factors tipping the balance between the two main TAM subsets. Applications derived from this report may help shape therapeutic strategies aimed at fortifying M1’s tumoricidal immunity to eradicate BC.

## 2. Tumor Microenvironmental Factors Influence Polarization of TAMs

### 2.1. Cytokines and Signal Pathways Interplay Between Malignant BC and TAMs Can Orchestrate M2 Polarization

#### 2.1.1. Colony Stimulating Factor 1 (CSF1) from BC Binds Receptor (CSF1R) on TAMs

Neoplastic cells promote TAM polarization towards the M2-like phenotype through various mechanisms. First, CSF1 secreted by BC cells attaches to CSF1R on macrophages and activates downstream signaling cascades responsible for the polarization of TAMs to immunosuppressive M2. In a study enrolled with 68 patients, high expression of CSF1 and CSF1R as well as high density of TAMs correlates with metastatic BC, among which CSF1 expression in malignant epithelial cells predicted BC mortality [38]. Hence, blockade of CSF1R using CSF1R inhibitor-loaded polymersomes enhanced the M2-to-M1 shift (Table 1) [39]. Moreover, dual treatment with a CSF1R inhibitor and the chemotherapeutic agent paclitaxel via pulmonary administration elevate M1 abundance while lowering tumor burden and lung metastasis [40]. On the other hand, CSF1-CSF1R interaction results in autophosphorylation of CSF1R which triggers phosphorylation and activation of downstream cascades, including the mitogen-activated protein kinase (MAPK) pathway, leading to proliferation and functional activation of M2 macrophages. Accordingly, delivery of dual kinase inhibitor-loaded supramolecular nanoparticles promoted M1 polarization (Table 1) [41].

#### 2.1.2. Crosstalk Between Signal Transducer and Activator of Transcription (STAT)3, IL-6, and Yes-Associated Protein (YAP) Pathways Regulate TAM Polarization

STAT3 has been demonstrated to be a crucial oncogenic signaling cascade exacerbating numerous cancers, including BC. Inhibition of STAT3 expression by the antisense oligodeoxynucleotide BP1003 not only enhances the sensitivity of HER2^+^ and TNBC to paclitaxel and 5-fluorouracil but also modulates the TME. Monocyte differentiation into M2 macrophages is remarkably attenuated by BP1003, indicating that STAT3 fosters M2 macrophage polarization; therefore, STAT3 abrogation may represent a potential immunotherapeutic strategy (Table 1) [42]. On the other hand, IL-6 secretion is augmented by multiple copies in T-cell malignancy 1 (MCT-1/MCTS1), an oncogene whose activation correlates with aggressive BC. Synergistically, they promote polarization of a monocytic cell line, THP-1, into the M2 subtype, thereby enhancing TNBC cell invasiveness. Conversely, MCT-1 knockdown has been shown to induce the tumor suppressor miR-34a, which alleviates IL-6 signaling and promotes higher M1 polarization [43]. In the context of IL-6, its suppression in conjunction with STAT3 inhibition by the naturally occurring polyphenolic compound resveratrol exerts tumoricidal effects by impeding cancer growth, enhancing chemosensitivity in BC cells, and elevating expression of the M1 marker C-X-C motif chemokine ligand 10 (CXCL10) along with an increased M1/M2 ratio (Table 1) [44]. In the BC immune milieu, STAT3 complexes with another oncogene, YAP, whose high expression in BC correlates with an elevated M2/M1 ratio. Given overexpression of YAP elevates IL-6 levels, co-expression of both not only is observed in aggressive basal-like BC but also correlates with enhanced stemness properties [45]. Moreover, dual inhibition of STAT3 and YAP decreased the M2/M1 ratio, elicited CD8^+^ and CD4^+^ T-cells, and enhanced T-cell proliferation, thereby restoring infiltration of anti-cancer immune cells [46].

The aforementioned findings have been substantiated in BC patients. In clinical studies, Wang et al. noted remarkably increased expressions of YAP, STAT3, and p-STAT3 in malignant core tissues compared to adjacent paratumor counterparts. Moreover, Western blot analysis revealed higher levels of YAP, STAT3, p-STAT3, vascular endothelial growth factor (VEGF), VEGFR-2, and PD-L1 as well as co-localization of YAP with M2 macrophages in the former than in the latter. Conversely, inhibition of YAP resulted in decreased STAT3, p-STAT3, PD-L1, VEGF VEGFR-2 expressions, thereby lowering the M2/M1 macrophage ratio and attenuating tumor growth in mice [46].

**Figure 1:**
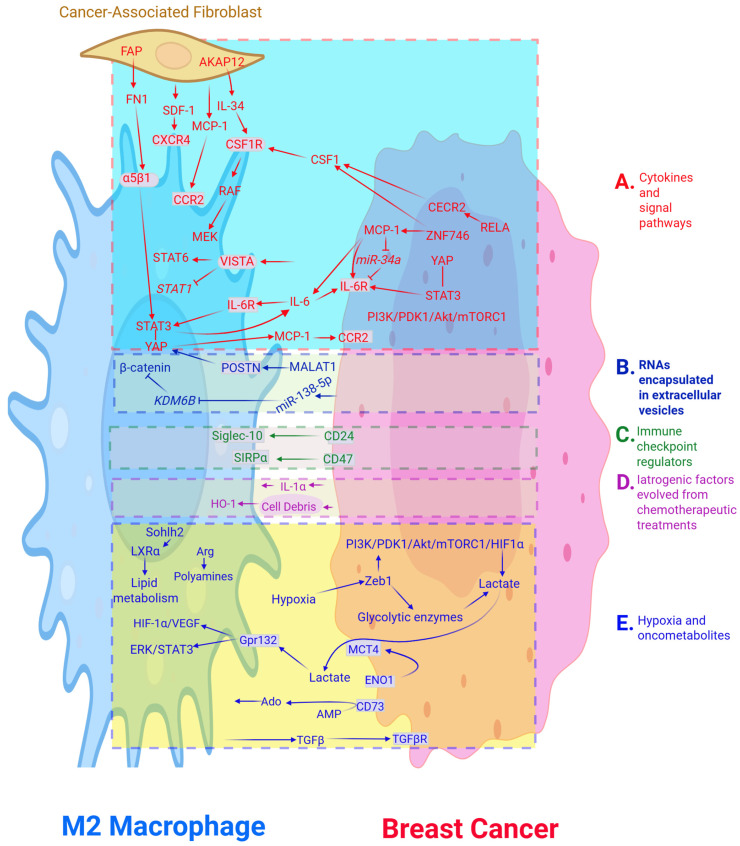
Modulators from breast cancer microenvironment maneuver M2 polarization. Cancer milieu consists of interacting breast cancer cells, the tumor-associated macrophage sub-type M2 and cancer-associated fibroblasts. Regulators steer M2 polarization are categorized into 5 groups (**A**–**E**). Fainted-head arrows with points or with blocked ends, respectively, indicate the activating or inhibitory impact on the targets (end of points). A solid-start arrow indicates conversion of arginine (Arg) to polyamines in the M2. A short line between the signal transducer and activator of transcription (STAT) and yes-associated protein (YAP) presents the binding between them. Cell surface proteins are in grey boxes, whereas darker shades highlight nuclei. While the regulators that favor neoplasms and promote M2 macrophages are in plain texts, the ones that attenuate cancer and augment M1 are italicized. Additional abbreviations used are denoted in the text body.

Furthermore, in the context of TAM polarization, STAT1 and STAT6 signaling cascades are regulated by V-domain immunoglobulin suppressor of T cell activation (VISTA) that serves as an inhibitory ligand or receptor that promotes immune tolerance. Accordingly, VISTA-deficient mice exhibited hindered tumor growth, accompanied by an increase in M1 and a decrease in M2 macrophages [47] (Figure 1A).

#### 2.1.3. Plasticity of TAMs Can Be Modulated by Signaling Cascades Comprising Phosphatidylinositol 3-Kinase (PI3K)/Phosphoinositide-Dependent Kinase 1 (PDK)1/Protein Kinase B (Akt)/Mammalian Target of Rapamycin Complex (mTORC)1 and Zinc Finger Protein 746 (ZNF746)

PDK1 was recognized to be crucial for tumorigenesis in spontaneous BC induced by polyoma virus middle T antigen [48]. Deletion of PDK1 in myeloid cells favors the polarization of macrophages toward the M1 phenotype in adipose tissue as well as the restoration of T cells and NK cells emanated from BC, thereby synergizing anti-tumor immunity [27]. Inhibiting its downstream signaling regulators, exemplified by Akt and mTORC1, yields tumor regression and ameliorates lung metastasis [27]. Likewise, suppression of its upstream modulator, namely PI3Kγ, using the inhibitor IPI-549 in combination with paclitaxel and immune checkpoint blockade (anti-PD1 antibodies) results in long-term BC remission and attenuates lung metastases. Triple-agent treatments alter the tumor immune landscape and manifest a prominent M2-to-M1 polarization; elevated CD4^+^, CD8^+^ T cells, B cells, and DC; as well as reduced regulatory T cell abundance and T cell exhaustion (Table 1) [49]. Moreover, expression of macrophage cytokines, such as MCP-1 and CSF1, can be augmented by ZNF746 that is highly expressed in BC cores compared with the surrounding non-cancerous tissues. Through activating the Jagged1/Notch cascade, ZNF746 positively correlates with metastasis and poor overall survival, as well as elevated M2 polarization, while its knockdown abolishes the aforementioned effects [50] (Figure 1A).

#### 2.1.4. Epigenetic Perturbations Influence TAM Polarization Through Aberrant Signaling Pathways

Dysregulated epigenetic modulators can skew the balance between M1 and M2. In this regard, one of the acetyl-lysine readers, cat eye syndrome chromosome region candidate 2 (CECR2), is recognized as an imperative upregulated epigenetic regulator in metastases, associated with an increased abundance of M2 macrophages and poor metastasis-free survival in BC. Mechanistically, among the nuclear factor kB (NF-kB) family members, v-rel avian reticuloendotheliosis viral oncogene homolog A (RELA) recruits CECR2 to increase chromatin accessibility and activate the expression of matrix metallopeptidase 2 (MMP2) and cytokine genes exemplified by CSF1 and chemokine (C-X-C motif) ligand 1 (CXCL1), which are crucial for immune suppression at metastatic sites. Hence, pharmacological abrogation of the CECR2 bromodomain not only downregulates CSF1 but also alleviates NF-kB-mediated immune inhibition and attenuated BC metastasis (Table 1) [51,52]. Among other epigenetic modulators, lysine demethylase 6B (KDM6B) contrarily induces macrophage polarization toward the anti-cancer M1 subset (Table 1) [53,54]. Hence, suppression of KDM6B expression in macrophages, by cancer cell-derived exosomal miR-138-5p, impedes M1 polarization while promoting M2 polarization. Macrophages treated with exosomal miR-138-5p exacerbate lung metastasis, and the quantity of circulating exosomal miR-138-5p positively correlates with poor prognosis in BC [53,54]. Du et al. ratified that KDM6B hinders M2 polarization via promotion of the intranuclear degradation of β-catenin that depends on the demethylase activity intrinsic to KDM6B. Its inhibition by miR-138-5p restores β-catenin/c-Myc signaling, whereas its induction by the active vitamin D analog paricalcitol results in cancer growth inhibition. Together, these findings suggest a novel therapeutic strategy consisting of the vitamin D supplement combined with epigenetic modulators [54] (Figure 1A and Figure 1B).

#### 2.1.5. Factors Evolved from Cancer-Associated Fibroblasts (CAFs) Favor M2 Polarization

CAFs have recently drawn growing attention for their roles as immune modulators, recruiting immune cells in addition to their intrinsic tumor-promoting functions. Mediated by stromal cell-derived factor-1 (SDF-1) and MCP-1, monocytes are recruited by CAFs to promote M2 polarization and immune tolerance [55]. On the other hand, A-kinase anchoring protein 12 (AKAP12), a member of the AKAP family, acts as a scaffold protein for signaling mediators as well as in the modulation of cell proliferation and migration [56]. The AKAP12^+^ CAFs subset is associated with immunotherapy resistance in TNBC, and their high abundance is correlated with poor prognosis [56]. Through activation of the PI3K/AKT/IL-34 axis, IL-34 is released from AKAP12^+^ CAFs, which binds to CSF1R on TAMs, thereby promoting polarization of the latter toward M2 phenotypes. Hence, pharmacological blockade of the IL-34/CSF1R signaling pathway improves the efficacy of targeted immunotherapy in TNBC rodent models (Table 1) [57]. Likewise, fibroblast activation protein (FAP) is overexpressed in a subset of CAFs denoted as FAP^+^ CAFs. They are associated with poor clinical outcomes and immunosuppression. FAP^+^ CAFs secrete fibronectin 1 (FN1) that crosstalks integrin α5β1 on macrophages to induce FAK-AKT-STAT3 signaling and then subsequently elicits polarization of the latter toward an immune inhibitory M2-like subfraction. Hence, pharmacological ablation of FN1–integrin α5β1 signaling using Cilengitide effectively abrogates immune escape effects in mouse models [58] (Figure 1A and Table 1).

### 2.2. Plasticity of TAMs Can Be Modulated by RNAs Encapsulated in EVs

Extracellular RNAs orchestrate TAM polarization through signaling cascades interplayed between metastasis-associated lung adenocarcinoma transcript 1 (MALAT 1)/periostin (POSTN), Hippo/YAP, and monocyte chemotactic protein-1 (MCP-1)/C-C motif cytokine ligand receptor 2 (CCR2) signaling cascades.

Crosstalk between distant cells involves the transfer of microRNAs, proteins, and lipids, all encapsulated in cargo-like structures known as EVs [59]. EVs orchestrate a variety of cellular and molecular pathways, resulting not only in physiological and pathological perturbations but also in neoplastic advancements [33,60]. In the latter scenarios, EVs exhibit immunosuppressive properties. For instance, TNBC-derived EVs shuttle the long non-coding RNA (lncRNA) MALAT1, which enhances M2 polarization and endows poor overall survival [61]. Likewise, in BC patients with positive hormone receptors or low tumor grade, meta-analysis showed that high expression of MALAT1 had a twofold higher risk of relapse compared to those with low expression [62]. Molecular studies revealed that in response to MALAT1 upregulation, the POSTN cascade is augmented, leading to increased M2 infiltration and poor prognosis in TNBC patients [62]. Moreover, the malignant properties of TNBC cells correspond to overexpression of another cancer modulator, YAP [63]. As one of the crucial components of Hippo signaling, YAP has been recognized as imperative for tumor formation, progression, metastasis, and immunity [63,64,65]. The linkage between MALAT1/POSTN and Hippo/YAP was unveiled by a study demonstrating that inhibition of the Hippo/YAP pathway abrogated the effect of POSTN overexpression on M2 marker expression (Table 1) [63]. On the other hand, MCP-1, formerly known as CC chemokine ligand 2 (CCL2), is produced by various cell types, including monocytes [66]. MCP-1 was significantly increased in YAP-overexpressing M2 macrophages compared with their YAP-low-expressing counterparts. The invasive properties of TNBC cells were significantly attenuated when the MCP-1/CCR2 signaling pathway was blocked in YAP-overexpressing M2 macrophages. These data suggest that YAP^+^ M2 macrophages promote TNBC cell invasion via MCP-1/CCR2 signaling (Table 1) [67]. Taken together, MALAT1 encapsulated in EVs secreted from TNBC and taken up by macrophages conspires with POSTN to elicit Hippo/YAP activation and provoke MCP-1/CCR2 signaling, leading to elevated M2 polarization [63,67]. Collectively, these mechanisms empower cancer metastasis, M2 polarization, and immune evasion (Figure 1A and Figure 1B).

### 2.3. Immune Checkpoint Blockades (ICBs) Maneuver TAM Polarization

Immune responses are modulated by a plethora of checkpoint regulators that act as “security stops” to pause immune reactions once an infection has ended, promote self-tolerance, and protect against autoimmunity. Hence, cancers exploit intricate immune checkpoint molecules to dampen host anti-tumor responses, evade immune recognition or destruction, and favor immune tolerance [68]. Accordingly, dysregulated checkpoint modulators promote M2 polarization [69]. Among them, cluster of differentiation (CD)24, a highly sialylated glycosyl-phosphatidyl-inositol cell surface protein, binds sialic acid-binding immunoglobulin-like lectin (Siglec)-10 on immune cells to elicit multifaceted roles in inflammatory diseases and in cancer progression (Table 1) [70]. In TNBC, sialylated CD24 on the surface of neoplastic cells serves as a “do not eat me” cue by binding to Siglec-10 on macrophages, thereby preventing cancer clearance and augmenting immune evasion [71]. Moreover, cancer cells express another checkpoint modulator, CD47, that exerts a distinct ‘‘engulf me not’’ signal that interacts with the signal regulatory protein α (SIRPα) receptor on macrophages to prevent phagocytosis [72]. This complex activates the Src homology region 2 domain phosphatases SHP1 and SHP2 in macrophages. Accordingly, blockade of both SHP2 and CSF1R by a dual-inhibitor-loaded nanoparticle yielded effective polarization of the M2 to the M1 phenotype, along with robust phagocytic capacity, compared with single-agent regimens (Table 1) [73]. Therapeutically, delivery of paclitaxel in conjunction with an anti-CD47 antibody (aCD47) via detachable immune liposomes (ILips) was developed to treat TNBC (Table 1) [74]. This ILip-mediated dual-agent strategy facilitates matrix metalloprotease 2 (MMP2)-responsive release of aCD47 to favor polarization of M2 macrophages toward the M1 phenotype, thereby enhancing engulfment of tumor cells [74].

Considering CSF-1 binds CSF-1R and then polarizes TAMs from a tumoricidal M1 phenotype to a pro-neoplastic M2 subtype, inhibition of CSF-1R in combination with deployment of SIRPα-blocking antibodies impairs both signaling cascades and enhances M2-to-M1 repolarization. This strategy also improves anti-tumor and antimetastatic efficacy compared with single-agent therapeutics [25,75,76]. Likewise, combinatorial blockade of the CD47/SIRPα and CD24/Siglec-10 axes by a bispecific antibody fusion protein enabled superior phagocytosis compared with agents that only blockade either CD47 or CD24 (Table 1) [77].

Moreover, hybrid cell membrane nanovesicles (known as hNVs) encompassing a SIRPα variant that mimics and hijacks the authentic CD47-SIRPα signaling were exploited, thereby weakening metastasis (Table 1) [78]. On the other hand, one of the major innate immune sensing pathways is identified to be a stimulator of interferon genes (STING). This signaling can be induced by vanillic acid through the STING/TBK1/IRF3 pathway which promotes macrophage polarization to the M1 phenotype via IL-6R/JAK signaling. Together, they synergistically enhance phagocytosis and apoptosis and inhibit cancer growth [79]. Therapeutically, an innovative agent consisting of a STING agonist incorporated into hNVs was developed to suppress cancer cell proliferation in a poorly immunogenic TNBC model [78]. Nevertheless, sensitivity to ICB can be boosted by introducing epigenetic modulators. For instance, entinostat, an oral histone deacetylase inhibitor, enhances responses to ICB in cancers with an immunosuppressive TME. Entinostat induces a shift from a pro-tumor to an anti-tumor TME, partly through epigenetic reprogramming of TAMs in HER2^+^ BC mouse models [80] (Figure 1C and Table 1).

### 2.4. Iatrogenic Factors Evolved from Post-Chemotherapeutic Treatments Augment TAM Polarization Towards M2

#### 2.4.1. Heme Oxygenase-1 (HO-1)

Through microtubule depolarization followed by cell growth arrest, paclitaxel incipiently acts as an antineoplastic agent and is widely used to treat a variety of solid cancers. Recently, paclitaxel was demonstrated to play a new role in promoting tumoricidal immunity. Via a TLR4-dependent manner, paclitaxel skews M2 to the M1-like phenotype [81]. However, this effect is diminished in paclitaxel-resistant milieu. Cytokine-enriched secretomes released from chemo-refractory cells shift macrophage polarization back toward the immunosuppressive M2 phenotype [82]. M2 polarization can be, however, sustained through the cancer-cell-derived debris generated upon use of chemotherapeutics, with involvement of another cancer-promoting modulator, namely HO-1 [83]. By metabolizing heme to carbon monoxide, biliverdin, and ferrous iron, HO-1 was initially recognized for its role in regulating anti-inflammatory responses, tissue defense, and antioxidative stress reactions [84]. Elevated HO-1 expression provokes chemoresistance of TNBC cells in response to doxorubicin treatments [85]. A total of 575 patients with locally advanced invasive BC were recruited to a clinical study. Results from survival analysis show that high HO-1 expression was significantly associated with poor disease-free survival (DFS) and overall survival (OS) of patients with TNBC [86]. Recently, Kim, et al. revealed that HO-1 promotes tumorigenesis and metastasis in many cancers, while its suppression facilitates M1 polarization [83]. HO-1 engages M2 polarization and enhances cancer recurrence in response to the malignant cell fragments generated post-chemotherapeutically, thereby restraining treatment efficacy [83]. Such a chemoresistant and cancer-promoting outcome can be abrogated by genetic ablation of HO-1 [87] (Figure 1D and Table 1).

#### 2.4.2. IL-1α

Immune cell-mediated neoplastic progression can be influenced by IL-1α released from malignant cells [88]. The mechanism by which IL-1α fosters immune tolerance was revealed in orthotopic transplantation murine models of BC, whereby loss of the host’s IL-1α gene led to the abortive rejection of the inoculated cancers [89]. IL-1α deficiency favored TAM polarization from M2 towards M1, which was accompanied by augmented cytotoxic T lymphocyte activity in the immune-competent TME. These lines of evidence underscore a pivotal role played by IL-1α in sculpting an immunosuppressive and cancer-promoting TME through TAM polarization, thereby implicating IL-1α as a promising target for eradicating BC [89]. Post-chemotherapeutically, dying necrotic BC cells release IL-1α that cultivates a myeloid-driven immunotolerant TME, inhibits CD8^+^ T cell recruitment and function, promotes tumor growth, and reduces survival [90]. Introducing a neutralizing IL-1α antibody reverses such neoplastic effects. Moreover, low IL1α expression correlates with promising prognoses in several solid malignancies, particularly in patients treated with chemotherapy (Table 1) [90]. Recently, Keerthi Raja et al. ratified that IL-1α promotes an immunosuppressive TME through reprogramming TAMs, thereby generating a pro-tumorigenic landscape [91] (Figure 1D).

### 2.5. Hypoxia and Oncometabolites Govern TAM Polarization

#### 2.5.1. Hypoxia Favors M2 Polarization

Hypoxia, a common feature of solid tumors, reprograms cancer cell metabolism, promotes immune evasion, and provokes drug resistance. The hypoxic cancer milieu has been unveiled as an inevitable factor governing M2 polarization and augmenting angiogenesis [92]. Hypoxia provokes Zeb1 activation that stimulates PI3K/Akt/hypoxia-inducible factor 1α (HIF-1α) signaling, leading to the accumulation of M2 and attenuating tumoricidal immunity, thereby conveying cancer recurrence and metastasis [93,94].

The results from an overall meta-analysis comprising 40 studies demonstrate that high HIF-1α expression is strongly linked to poor outcomes in BC patients. Furthermore, both OS and DFS were markedly shorter in TNBC cases with high HIF-1α expression than the ones with low expression [95].

Under the hypoxic TME, M2 macrophages accumulate in peri-necrotic regions, where placental growth factor and its ligand, vascular endothelial growth factor (VEGF) receptor-1, are upregulated. Hence, introducing the HIF-α inhibitor YC-1 (lificiguat) not only induces TAM polarization towards the M1 phenotype but also diminishes angiogenesis and impedes cancer growth (Table 1) [96]. Likewise, application of a hollow gold–platinum bimetallic nanoshell in BC inspires a significant increase in oxygen levels and marked cancer regression through photothermal impacts. The raised oxygen levels alleviate tumor hypoxia, shift M2 into M1 macrophages, weaken HIF-1α, dampen multidrug resistance gene 1, and facilitate the release of doxorubicin, thereby restoring immune sensitivities and improving chemotherapy efficacy [97] (Figure 1E and Table 1).

#### 2.5.2. Lactate Metabolism Governs M2 Polarization

Polarization of macrophages strongly correlates with their metabolic landscapes. Oncometabolites in the TME constitute a major obstacle to anti-tumor immunity [98]. Strategies to reprogram TAMs are emerging based on the finding that different TAM subtypes exhibit distinct metabolic networks [99]. Aerobic glycolysis has been unveiled to facilitate tumor progression by producing lactate which acts as an anti-inflammatory, pro-tumorigenic, and immunosuppressive mediator [94]. Among the glycolytic enzymes, enolase 1 (ENO1) is involved in macrophage plasticity and neoplastic progression in BC. Induced by oncogenic TGFβ1/Smad3 signaling and mediated through protein arginine methyltransferase 5, the arginine moiety on ENO1 becomes demethylated, enabling translocation of ENO1 on the membrane, thereby interacting with monocarboxylate transporter 4 (MCT4) for lactate secretion, recruiting M2 macrophages, and promoting an immunosuppressive TME [100]. Lactic acid activates the G protein-coupled receptor 132 (Gpr132) signal transduction on TAMs, thereby exacerbating cancer metastasis and angiogenesis [101]. Accordingly, high Gpr132 expression favors metastasis and poor prognosis in patients with BC, whereas its deletion not only hinders M2 polarization but also attenuates BC lung metastasis in mice [101]. For M2 polarization, the lactate/Gpr132 axis interplays with ERK1/2/STAT3 and HIF-1a/VEGF signaling cascades [102,103]. Thus, introducing inhibitors such as selumetinib and stattic, which respectively block ERK and STAT3, not only diminished lactate-induced M2 polarization but also inhibited proliferation, migration, and angiogenesis of cancer cells [103]. Similarly, the natural compound withanolide D substantiates the evidence by demonstrating that the ERK/STAT3 signaling blockade represents a promising therapeutic strategy. These findings corroborate the claim that the lactate–ERK/STAT3 signaling pathway strengthens BC progression through M2 polarization [103] (Table 1).

As BC progresses, the hypoxic TME augments glycolytic activities by upregulating the PI3K/Akt/HIF-1α signaling axis through Zeb1 activation. Hence, ectopic expression of Zeb1 directly increases the transcripts of glycolytic rate-determining enzymes, namely HK2, PFKP, and PKM2, thereby augmenting glycolytic activity and elevating lactate production, which cultivates an immunosuppressive TME, promotes M2 polarization, and ultimately aggravates cancer progression [94]. In concordance, the expression of the lactate transporter SLC16A3 is correlated with highly proliferative BC molecular subtypes [15]. The abundance of M1 macrophages is reduced in BC with SLC16A3 expression. Likewise, in vitro experiments ratified that lactate could hinder the expression of M1 markers [15]. Taken together, lactic acid produced by cancer cells promotes the polarization of TAMs towards M2, while obscuring the expression of M1 markers, thereby weakening innate immune defenses and promoting cancer progression [15,104].

#### 2.5.3. Arginine Metabolism Regulates M2 Polarization

The ratio of M1/M2 TAMs is demarcated by differential arginine metabolism, in which M1 converts arginine to nitric oxide (NO), whereas M2 metabolizes arginine to polyamines that foster a tumorigenic TME [18,105]. The scenario occurring in the latter is attributed to aberrant NO synthase activity caused by oxidative degradation of the essential enzyme cofactor tetrahydrobiopterin (BH4), triggered by activated ERBB2 and TGFβ signaling cascades [105]. Hence, replenishing the endogenous BH_4_ precursor, known as sepiapterin (SEP), restored BH_4_ levels in M2-like macrophages, shifted arginine metabolism to NO synthesis, and then polarized M2 to type M1. The reprogrammed M1 manifested full-blown activities of antigen presentation and effector T induction for eliciting immunogenic cell death of HER2^+^ BC cells. This study ratifies a promising application of SEP by countering oncometabolites [18].

#### 2.5.4. Adenosine Generated by Cluster of Differentiation (CD)73 Orchestrates M2 Polarization

Prolonged inflammation, hypoxia, and anti-cancer drug treatments can elevate aberrant CD73 expression, which is highly correlated with TAM plasticity, immune evasion, and drug resistance [106]. CD73 is an enzyme that generates extracellular immunosuppressive adenosine via enzymatic dephosphorylation of adenosine monophosphate. Its expression is correlated with an immune-tolerant TME and poor prognosis in TNBC [107]. High CD73 expression in BC was associated with the development of bone metastases in a large, academic, multi-center, randomized phase-III study on a cohort enrolled with 3300 BC patients [108]. On the other hand, transforming growth factor (TGF) β is a distinct notorious immunosuppressive cytokine that synergizes with CD73 to further aggravate BC [109] (Table 1). TNBC treated with a dual functional anti-CD73-TGFβ construct consisting of the CD73 antibody MEDI9447 fused with the TGFβRII extracellular domain (termed MEDI-TGFβR) yielded diminished levels of M2-like TAMs, along with substantially elevated levels of activated DC, cytotoxic T cells, B cells and accumulation of M1 macrophages in a syngeneic mouse model of TNBC [109].

#### 2.5.5. Lipid Metabolism Steers M2 Polarization

Exposure to lipopolysaccharides associated with microbials augments M1 polarization, increases ROS production, and promotes phagocytosis [110,111]. Contrarily, M2 macrophages not only maintain lower levels of ROS but also exhibit robust fatty acid oxidation (FAO) [112,113]. Inhibition of FAO provokes anti-tumor cytotoxicity and stifles cancer growth [114]. Mounting evidence suggests that the tumor–adipose microenvironment is a universal microecosystem characterized by excessive free fatty acids [115]. A plethora of human cancers, including BC, retain high levels of lipid metabolites [113,116,117,118]. Lipid accumulation favors mitochondrial FAO as a primary energy source in TAMs, thereby not only sustaining them in the M2 subset but also suppressing inflammation and cytotoxicity exerted by innate immunity [119]. Furthermore, a positive correlation between lipid metabolism and M2 abundance closely coincides with the involvement of obesity in activating M2 macrophages [120,121].

Aberrant lipid metabolism underlying M2 polarization has been demonstrated. One of the transcription factors, namely Spermatogenesis- and oogenesis-specific basic helix-loop-helix (Sohlh2), is dysregulated in lipid metabolism. Molecular studies revealed that by directly binding to the promoter of liver X receptor α (LXRα) and enhancing its transcription activity, Sohlh2 upregulates LXRα expression, thereby skewing lipid homeostasis on the membrane of macrophages and favoring M2 polarization [122]. Thus, high expression of Sohlh2 in M2 macrophages enhanced TNBC cell growth, migration, and lung metastasis both in vivo and in vitro. Moreover, upon arriving in lipid-rich lymph nodes, metastatic malignancies undergo metabolic reprograming toward FAO. Hence, delivery of the metabolism-regulating drug etomoxir to cancer-draining lymph nodes not only corrects FAO dysregulation at metastatic sites but also attenuates M2 polarization [123] (Table 1). Along with this notion, internalization of immunometabolic nanoparticles (immeNPs) by TAMs reprograms lipid metabolism, results in innate immune stimulation, promotes M2-to-M1 repolarization, as well as elevates infiltration of immunogenic DC and cytotoxic T cells [124] (Table 1). Likewise, inhibition of hedgehog signaling in M2 attenuated FAO and yielded macrophages reminiscent of the M1 subtype [125] (Table 1).

## 3. Advancements in TAM Studies

TAMs are recognized to be more heterogeneous than a strict binary system. Based on gene expression profiles, active metabolic pathways, and functions associated with neoplastic characteristics, continuum subgroups of TAMs have been identified, even though they can still be largely categorized as M1-like or M2-like [31,34,126,127]. Furthermore, a cutting-edge cell lineage tracing technology via single-cell RNA transcriptomics has demonstrated that human TAMs are diverse in origin, including both resident tissue macrophages (RTMs) and monocyte-derived macrophages from the bone marrow [128,129]. Moreover, the same technology revealed that there is no available biomarker that is uniformly expressed on M1- or M2-like macrophages, reflecting a limitation of the classical paradigm [130]. TAMs from BC are transcriptionally distinct from monocytes and respective tissue-resident macrophages, indicating that the former embarks transcriptional reprogramming throughout BC neoplasms [130]. Likewise, correlating transcriptomics with the distribution of TAMs in the TME sheds new insights into TAM polarization. For example, monocyte-derived TAMs tend to localize in the core of the tumor mass, while RTMs are more abundant in the peripheral margines [129], suggesting the former are likely to polarize more toward M2-like TAMs, while the latter are M1-like.

Due to the immense collection of omics and spatial imaging integrated with machine learning algorithms, artificial intelligence has promptly gained a good standing in TAM research [131]. For instance, combining laboratory and clinical data uncovered a previously unidentified TAM subpopulation that is particularly associated with poor OS and chemotherapy resistance in BC patients [132].

## 4. Pitfalls of TAM-Targeting Therapies

In 2005, Guiducci et al. reported an unprecedented succuss engaging a macrophage-targeting therapy in murine BC models [133]. The study utilized intratumoral delivery of a CCL16-expressing recombinant adenovirus to mice, followed by intravenous injection of a CpG oligodeoxynucleotide (CpG-ODN) and anti-IL10R antibodies. The strategy was to recruit and reprogram macrophages toward tumoricidal M1-like TAMs. Outcomes from this approach were promising, indicated by the finding that more than 60% of mice aborted the BC inoculum and sustained cancer-free survival [133]. However, over a decade later, treatment regimens cannot be recapitulated in clinical trials, except that the CSF1R inhibitor pexidartinib has been approved by the US Food and Drug Administration (FDA) to treat tenosynovial giant cell tumor, albeit its effects on cancer remain disappointing [134,135]. Emerging TAM-targeting agents in clinical trials have been systematically reviewed [136]. Here we articulate how challenges may evolve.

### 4.1. Low Specificity Results in Off-Target Side Effects

The majority of cytokines that steer macrophage polarization, such as CSF1, MCP-1, and IL-6, are broadly engaged in diverse physiological processes. IL-6 inhibition protocols have been introduced to treat iatrogenic side effects arising from treatments with immune checkpoint inhibitors [137]. However, no direct therapeutic effects on cancer have been observed in large clinical trials [138]. On the other hand, even though several MEK inhibitors and a PI3K inhibitor have been approved for cancer treatments by the FDA, their primary mechanism of action is not macrophage reprogramming but rather direct inhibition of cancer cell proliferation. Inevitably, involvement of pleiotropic physiological networks by drug targets often results in adverse side effects. For example, CD47 is ubiquitously expressed in human cells as a “do not eat me” signal to dysregulate autoimmunity [139]. Antibodies blocking CD47 have resulted in a wide range of side effects, including severe anemia. The phase-3 trial of a CD47-targeting agent, magrolimab, was terminated in 2023 due to side effects, while the phase-2 trial of an improved agent, maplirpacept, with minimal erythrocyte binding was discontinued in 2025, citing difficulty in recruiting subjects [140]. Contrarily, CD24 is expressed in lower levels in normal tissues such that anti-CD24 regimens are better tolerated [141,142].

To mitigate side effects, a logical approach is to target receptors or ligands that are less abundant but restricted to TAMs. SIRPα, the ligand of CD47, is mostly limited to the myeloid lineage [143], whereas siglec-10, the ligand of CD24, is largely restricted to immune cells [144]. Targeting SIRPα, via either a high-affinity decoy or monoclonal antibodies, has resulted in better tolerance than antibodies against CD47 [145,146]. Meanwhile clinical trials of a monoclonal antibody against Siglec-10 are ongoing. It is worth mentioning that for antibody therapies, low specificity also predicts poor efficacy, due to the fact that off-tumor targets comprise “antigen sink” that sequesters antibodies. This may have accounted for lower responses from anti-CD47 therapy compared to anti-SIRPα regimens [147,148]. Nevertheless, advancements in TAM biology may shed light on identifying TAM-specific therapies that spare normal macrophages.

### 4.2. Compensatory Feedback Pathways Confer Unsatisfied Efficacy in Treatment Regimens

Other obstacles impeding the targeting of M2-polarizing factors include pathway redundancy, compensatory feedback, and negative feedback. For instance, both CSF1 and IL-34 stimulate CSF1R, while both CD24 and CD47 suppress phagocytosis. Targeting both CD24 and CD47 using a bispecific antibody resulted in more robust anti-cancer efficacy than either single agene alone [77]. Within the same pathway, inhibiting one factor may be compensated by overexpression of another. When RAF or MEK is inhibited in melanoma cells, levels of CSF1 and IL-34 become elevated [149]. Similar compensation mechanisms are noted in TAMs. Cancer patients receiving pexidartinib, a CSFR1 inhibitor, demonstrated increased levels of CSF1 in circulating plasma [150]. In a mouse model inoculated with the BC cell line 4T1, arginine starvation induced a compensatory increase in IFNγ-JAK-STAT signaling in both tumor cells and in TAMs [151].

Negative feedback circuits are common components of normal immune responses to maintaining a dynamic balance between inflammation and tissue healing as well as the magnitude and duration of inflammation. Hence, the natural physiologic circulatory loop could limit the outcomes of immunostimulatory interventions. For example, CpG-ODN not only stimulates Toll-like receptor (TLR) 9 in murine macrophages to promote M1 polarization but also induces IL-10 production to counter this effect [152]. Accordingly, dual therapy using CpG-ODN and anti-IL-10R favors M1 macrophages and inhibits tumor growth [153,154]. Similarly, chronic activation of STING eventually causes upregulation of immune checkpoint molecules [155]. Nonetheless, combinatorial therapies are pivotal for successful TAM-targeting therapeutics.

### 4.3. Leaping Translation and Knowledge Gaps Prior to Achieving Clinal Efficacies

Fundamental differences between human and murine macrophages exist. Murine macrophages express higher TLR9 compared to human counterparts [156]. Likewise, while IL-4 and IL-13 are the main drivers of M2 polarization in mice, their roles in human macrophage activation remain unelucidated [157]. Variations in upstream inducers result in different landscapes in TAM subsets. Some key markers for M1 and M2 macrophages differ between mice and humans [19]. While a dichotomous classification of M1 and M2 is exemplified in mouse models, there is a continuum between M1 and M2 of human macrophages with cells expressing markers of both subtypes [158]. Nonetheless, discrepancies in the aspects of macrophage biology, the TME, and pharmacokinetics could predict promising outcomes generated from TAM-Targeting treatments in mouse models may result in low responses in human studies.

Macrophage differentiation and polarization in humans remain enigmatic. Several classification algorithms have evolved subsets of TAMs, ranging from four to seven, based on the context of gene signatures, surface markers, and cytokine profiles [159,160]. Among them, complement component 1q (C1Q)+ TAM and secreted phosphoprotein 1 (SPP1)+ TAM are the two main subpopulations across diverse cancer types. Although both subtypes may express the M2-like markers CD163 and CD206, pathophysiological functions and prognostic significance in different malignancies are inconsistent [159]. C1Q+ TAM is likely developed from monocytes via an M1-like intermediate, while SPP1+ TAM appears to originate from both monocytes and RTMs. Nevertheless, their exact evolutionary pathways throughout different cancer stages are largely ambiguous [159]. C1Q+ TAM expresses high CSF1R in colorectal cancer [161] and shows enhanced fatty acid metabolism in malignant pleural effusion [162]. Yet, the sensitivity to CSF1R inhibitors and metabolic modulators across cancer types are not well characterized. Similarly, the SPP1+ TAM subset is susceptible to treatment by an SPP1 inhibitor, namely nilotinib, which may be responsible for exerting an anti-cancer effect in ovarian cancer [163]. However, nilotinib is a nonspecific tyrosine kinase inhibitor originally developed to target other signaling pathways in chronic myeloid leukemia [164] and is effective in treating Parkinson’s disease [165]. Hence, its anti-cancer outcome may not be solely due to TAM repolarization but rather multifaceted effects. Nonetheless, identifying and suppressing M2-promoting targets for treating human BC still await further investigations.

## 5. Conclusions and Future Directions

It has been broadly recognized that the complex crosstalk between BC and the immune milieu determines whether neoplastic cells evade surveillance or are eradicated. The warfare between tumoricidal and neoplastic-promoting responses centers on the plasticity and heterogeneity of TAMs. Acting like a double-edged sword, macrophages incipiently consist of a proinflammatory tumoricidal M1 subpopulation but further advance to a cancer-promoting subset (M2), thereby contributing to multiple aspects of BC progression. Despite the fact that anti-TAM therapies across BC remain elusive to date, future effective TAM-targeting therapies should address not only reprogramming TAMs toward the M1 subset or attenuating the M2 subgroup via singular or combinatorial targets (exemplified in Table 1) but also fulfilling knowledge gaps and addressing the differential biology as well as cytotoxicity and pharmacokinetics between mice and humans that are pivotal to overcome translational obstacles.

**Table 1:** Potential therapeutic targets abrogate M2 polarization in breast cancer.

Therapy	Target	BC Subtype Studied	Rationale	Reference(s)
CSF1R inhibitor	CSF1R	TNBC	CSF1 augments polarization of TAMs toward M2.	[39]
CSF1R/MAPK dual inhibition	CSF1R and MAPK	TNBC	MAPK transduces CSF1R signal.	[41]
Antisense oligodeoxynucleotide	STAT3	HER2^+^ and TNBC	STAT3 transmits IL-6 signal favoring M2 polarization.	[42]
Resveratrol	IL-6 and STAT3	TNBC	IL-6 activates STAT3 and induces M2 polarization.	[44]
IPI-549	PI3K-γ	Luminal B	PI3K-γ activates PDK1/Akt/mTORC1 in M2.	[27,49]
NVS-CECR2–1 Bromosporine	CECR2	TNBC	CECR2 is abrogated to hinder M2 and metastasis.	[51,52]
Paricalcitol	KDM6B	TNBC	Restoration of KDM6B promotes M1 polarization epigenetically.	[54]
Anti-IL-34 antibody	IL-34-CSF1R signaling	TNBC	IL-34 stimulates CSF1R to promote M2 polarization.	[57]
Cilengitide	Ablation of FN1–integrin α5β1	TNBC	FN1-α5β1 induces M2 polarization.	[58]
Verteporfin	YAP	TNBC	YAP executes MALAT1-induced M2 polarization.	[63]
MCP-1-neutralizing antibody or RS102895	MCP-1 and CCR2 respectively	TNBC	MCP-1/CCR2 pathway blockade attenuates BC invasiveness.	[67]
Anti-CD24 antibody	CD24	TNBC	CD24 binds Siglec-10 on macrophages to suppress phagocytosis.	[70]
SHP099	SHP2	TNBC	SHP2 conveys CD47-SIRPα antiphagocytic signal.	[73]
Anti-CD47 antibody	CD47	TNBC	CD47 binds TAMs to inhibit phagocytosis.	[74]
High-affinity SIRPα variant with cGAMP in nanovesicles	Hijacks SIRPα on TAMs with STING	TNBC	Blockade of CD47-SIRPα signaling alleviates checkpoint modulators as cGAMP activates STING/IRF3 to favor M1.	[78]
Anti-SIRPα antibody	SIRPα	TNBC	CD47-SIRPα abrogated signaling to inactivate checkpoint regulators.	[76]
Anti-CD24/anti-CD47 bispecific antibody	CD24 and CD47	TNBC	Dual blockades for antiphagocytotic signals.	[77]
Entinostat	HDAC	HER2^+^	Epigenetic reprogramming of TAMs.	[80]
ZnPPIX	HO-1	TNBC	HO-1 favors M2 polarization.	[84,87]
Anti-IL-1α antibody	IL-1α	Luminal B	IL-1α promotes M2.	[90]
YC-1	HIF-1α	TNBC	HIF-1α promotes glycolysis, angiogenesis, and immune tolerance.	[96]
Bimetallic nanoshell	hypoxia	TNBC	Pt-catalyzed oxygen generation hinders HIF-1α, aside from photothermal therapy and enhanced drug delivery.	[97]
Selumetinib	ERK	TBNC	ERK favors M2 polarization in response to lactate accumulation.	[103]
Stattic	STAT3	TNBC	STAT3 is pivotal for M2 polarization and multiple processes of cancer development.	[103]
Withanolide D	ERK/STAT3	TNBC	Both targeted modulators are imperative for M2 polarization.	[103]
MEDI-TGFβR	CD73 and TGFβ	TNBC	Simultaneous inhibition of TGFβ and CD73 signaling to attenuate immunosuppressive adenosine.	[109]
Etomoxir	Carnitine Palmitoyltransferase-1	TBNC	Diminished key enzymes involved in FAO.	[123]
immeNPs	Reprogram fatty acid metabolism	TBNC	Attenuation of FAO to induce M2-to-M1 shift.	[124]
Vismodegib	Hedgehog	TBNC	Inhibition of FAO promotes M1.	[125]

Table legend: For abbreviations shown in this table, refer to the text body and the legend in Figure 1.

## Data Availability

No new data were created or analyzed in this study. Data sharing is not applicable.
